# Asthma and Pneumonia among Children Less Than Five Years with Acute Respiratory Symptoms in Mulago Hospital, Uganda: Evidence of Under-Diagnosis of Asthma

**DOI:** 10.1371/journal.pone.0081562

**Published:** 2013-11-29

**Authors:** Rebecca Nantanda, James K. Tumwine, Grace Ndeezi, Marianne S. Ostergaard

**Affiliations:** 1 Child Health and Development Centre, Makerere University College of Health Sciences, Kampala, Uganda; 2 Department of Paediatrics and Child Health, Makerere University College of Health Sciences, Kampala, Uganda; 3 The Research Unit for General Practice and Section of General Practice, Department of Public Health, University of Copenhagen, Copenhagen, Denmark; Kliniken der Stadt Köln gGmbH, Germany

## Abstract

**Background:**

Pneumonia is considered the major cause of mortality among children with acute respiratory disease in low-income countries but may be over-diagnosed at the cost of under-diagnosing asthma. We report the magnitude of asthma and pneumonia among “under-fives” with cough and difficulty breathing, based on stringent clinical criteria. We also describe the treatment for children with acute respiratory symptoms in Mulago Hospital.

**Methods:**

We enrolled 614 children aged 2–59 months with cough and difficulty breathing. Interviews, physical examination, blood and radiological investigations were done. We defined asthma according to Global Initiative for Asthma guidelines. Pneumonia was defined according to World Health Organization guidelines, which were modified by including fever and white cell count, C-reactive protein, blood culture and chest x-ray. Children with asthma or bronchiolitis were collectively referred to as “asthma syndrome” due to challenges of differentiating the two conditions in young children. Three pediatricians reviewed each participant’s case report post hoc and made a diagnosis according to the study criteria.

**Results:**

Of the 614 children, 41.2% (95% CI: 37.3–45.2) had asthma syndrome, 27.2% (95% CI: 23.7–30.9) had bacterial pneumonia, 26.5% (95% CI: 23.1–30.2) had viral pneumonia, while 5.1% (95% CI: 3.5–7.1) had other diagnoses including tuberculosis. Only 9.5% of the children with asthma syndrome had been previously diagnosed as asthma. Of the 253 children with asthma syndrome, 95.3% (95% CI: 91.9–97.5) had a prescription for antibiotics, 87.7% (95% CI: 83.1–91.5) for bronchodilators and 43.1% (95% CI: 36.9–49.4) for steroids.

**Conclusion:**

Although reports indicate that acute respiratory symptoms in children are predominantly due to pneumonia, asthma syndrome contributes a significant proportion. Antibiotics are used irrationally due to misdiagnosis of asthma as pneumonia. There is need for better diagnostic tools for childhood asthma and pneumonia in Uganda.

## Introduction

Pneumonia has hitherto been regarded as the major cause of respiratory morbidity and mortality among children less than five years in low-income countries [1,2]. Evidence suggests that pneumonia in this age group may be over-diagnosed and therefore its prevalence over-estimated [3,4]. In Uganda, pneumonia is the third most common cause of morbidity among children less than five years [5]and is associated with high case fatality rates ranging from 10 to 30% [6–8]. A recent study done in Uganda showed that only 6.1% of the children who presented with cough and/or difficult breathing were diagnosed with asthma [9].

Asthma is the most common chronic childhood condition worldwide [10,11]. Asthma exacerbations may have clinical presentation similar to pneumonia [10,12]. According to World Health Organization (WHO) guidelines on Integrated Management of Childhood Illnesses (IMCI), diagnosis of asthma is based on presence of audible wheeze [13]. However, audible wheeze has low sensitivity for diagnosis of asthma. It is present in only one-third of children with auscultatory wheeze [3,14]. In addition, the symptom of wheeze may not be easily recognized by caretakers [15,16].

 In young children, asthma and bronchiolitis are closely related in aetio-pathogenesis and clinical presentation [12,17]. It is therefore difficult to distinguish asthma from bronchiolitis, especially among children less than 2 years of age. There is no common international definition for bronchiolitis, but generally, it is defined as the first episode of severe wheezing in association with cough and/or difficulty breathing among children less than 12-24 months of age [18]. On the other hand, asthma among children less than five years is well described [19]. It is defined as a chronic inflammatory disorder with hyper-reactive airways resulting in cough, recurrent wheeze, breathing difficulties and chest tightness, particularly at night and/or early morning [20]. In this study, we use the term “asthma syndrome” to refer to children who had asthma or bronchiolitis. Using the term “asthma”, which is recurrent and potentially remittent, affords an opportunity for children with asthma syndrome to under-go regular diagnostic re-evaluation and eventual identification of children with asthma. Studies have indicated that about 30-50% of children who are diagnosed with bronchiolitis in early childhood develop asthma later on [21,22].

According to IMCI guidelines, pneumonia is diagnosed in presence of cough and/or difficulty breathing, and fast breathing, with/without lower chest in-drawing where the chest moves in or retracts during inspiration. However, previous studies indicate that some children with similar signs have viral pneumonia [23–25]. In this study, children who had tachypnoea with or without chest retractions but did not fulfill the criteria for bacterial pneumonia were classified as viral pneumonia. 

Over-diagnosis of pneumonia and under-diagnosis of asthma may be contributing to significant untreated respiratory morbidity and mortality among children less than five years in low-income countries. This could hinder the anticipated progress to achieving Millennium Development Goal 4 (MDG 4)-to reduce child mortality by two-thirds by the year 2015. The aim of this study was to determine the magnitude of asthma and pneumonia among children less than five years of age with cough and/or difficulty breathing, based on stringent clinical criteria. We also describe the treatment for children with acute respiratory symptoms in Mulago hospital Uganda.

## Methods

### Ethical statement

The study was approved by the Higher Degrees, Ethics and Research Committee (HDREC) at Makerere University, College of Health Sciences and Uganda National Council of Science and Technology (UNCST). Informed written consent was obtained from the caretakers of the participants. Additional consent was sought for HIV testing. Approval to extract prescription notes from patient files was obtained from the Department of Paediatrics and Child Health, Mulago hospital.

### Design and Setting

A cross-sectional study involving 614 children aged 2 to 59 months presenting at the emergency paediatric unit of Mulago Hospital, Kampala, was conducted between August 2011 and July 2012. Mulago Hospital is a National Referral Hospital in Uganda and a teaching hospital for Makerere College of Health Sciences, Kampala. It also acts as a district hospital serving an urban and peri-urban catchment population of about two million people. Most services at the hospital are paid for by the state. The paediatric emergency unit is comprised of the paediatric intensive care unit (PICU) and, high and low-dependency wards. Critically ill children aged 1 day up to 12 years of age are admitted at the unit and transferred to other wards upon stabilization. The average daily attendance is 80 children, 75% of whom are aged 2 to 59 months. An estimated 25% of the children present with cough and/or difficulty in breathing. The hospital was selected as the study site because of its ability to handle laboratory and radiological investigations for asthma and pneumonia, services that are not readily available in other public hospitals in Uganda.

### Data collection procedure

 All children attending the paediatric emergency unit at Mulago hospital were screened and those aged 2-59 months with cough and/or difficulty in breathing identified. Children with fast breathing and/or chest retractions were included. Fast breathing was defined as ≥50 breaths per minute for children aged 2 up to 12 months and ≥40 breaths per minute for children aged 12 up to 59 months. We excluded children with cardiac failure secondary to severe anaemia or heart disease. After obtaining informed written consent from the caretakers of the children who fulfilled the inclusion criteria, a questionnaire was administered by the nurse. This was followed by a clinical examination by the study doctor. Children with severe classification [26] were stabilized before proceeding with consent. Peripheral oxygen saturation (SaO_2_) in room air was measured and for those children whose SaO_2_ was <92%, oxygen was given using mask, catheter or nasal prongs. Children with wheezing were given salbutamol solution (2.5mg in 3-5mls of normal saline) using an ultrasonic nebulizer according to the hospital protocol [27]. The response to the nebulized salbutamol with respect to the respiratory rate and presence and intensity of wheezing was noted. Nutritional assessment was done according to WHO guidelines on management of children with severe malnutrition [28]. Six mill-liters (6mls) of venous blood were drawn from the cubital vein or dorsum of the hand using a BD™ blood collection set and used for blood culture, white cell count, serum C-reactive protein (CRP) and HIV tests. A peripheral blood smear for malaria parasites was also done. A specimen of nasopharyngeal epithelium was collected for identification of Respiratory Syncytial Virus (RSV) in accordance with manufacturer’s instructions (BD Diagnostics, Becton, Dickinson and Company, USA). All specimens were transported to the respective laboratories within 6 hours of collection. The details of the laboratory methods have been described elsewhere [29]. A posterior-anterior chest x-ray was taken within 48 hours after recruitment. The data was shared with the Department of Paediatrics and Child Health, Mulago National Referral and Teaching Hospital.

### Management of study participants

The ward doctors were primarily responsible for management of the study participants. However, the study team ensured that all participants who needed short-acting bronchodilators received the recommended three doses and noted the response. Information on the patients’ prescription was extracted from their files in order to understand how ward doctors manage the children with acute respiratory symptoms. The study team communicated the test results of the participants as soon as they were obtained.

### Definitions

The study definitions were formulated based on current international guidelines and in consultation with experts as follows:

#### Asthma

We used a modified definition of asthma derived from the Global Initiative for Asthma (GINA) guidelines [19]. We made the following modifications; we excluded the symptom of “chest tightness” because young children are not able to express this symptom objectively [30,31]. We also excluded measurement of peak flow and/or spirometry because children less than five years are not able to perform these tests effectively [32]. Furthermore, we included chest x-rays to help us differentiate asthma and pneumonia. Pneumonia is very common in Uganda and among children less than five years, the clinical presentation of acute asthma and pneumonia can be very similar [5,12].

**Bronchiolitis** was defined as the first episode of wheezing in a child less than 24 months of age, presenting with cough and/or difficult breathing, and respiratory distress. This was based on the South African guidelines for diagnosis, management and prevention of acute viral bronchiolitis [18]. 

#### Asthma syndrome

In this study, children with asthma or bronchiolitis were collectively referred to as “Asthma syndrome”. Cohort studies on genesis and progression of childhood asthma noted that asthma symptoms start in early childhood [33]. However, among young children, it is difficult to distinguish acute asthma and bronchiolitis because of the similarities in clinical presentation [12]. Many terminologies including bronchitis, bronchitis-asthma, wheezing disorder and hypereactive airways disease, have been used to describe children with cough and wheezing [12]. We decided to use the term “asthma syndrome” to include all children with cough and wheezing, because such symptoms can be controlled with available medicines. Furthermore, an asthma-related diagnosis would indicate the potential for follow up and diagnostic re-evaluation. Through this process, children with asthma would be identified early and hence benefit from the available safe and effective asthma medicines which would contribute towards improvements in their quality of life.

#### Pneumonia

We defined pneumonia as presence of cough and/or difficult breathing and fast breathing, with/without lower chest in-drawing. However, studies among children with WHO-defined pneumonia have indicated that not all of them have bacterial aetiology [23,34]. Therefore the following modifications were made to improve on the specificity of this definition;

1 We included results of chest x-ray to help distinguish viral and bacterial pneumonia in some children. We acknowledge that chest x-ray findings alone cannot be used to differentiate viral from bacterial pneumonia [35,36]. However, in some cases, the chest x-ray findings imply a particular aetiology. For example, consolidation and pneumatoceles are usually associated with bacterial pneumonia [35,37].
2 We also included test results for white cell count (total and differential), blood culture and serum C-reactive protein, to help differentiate viral from bacterial pneumonia. Although these tests do not explicitly identify the aetiology of pneumonia, when used in combination with history and examination findings can help to identify the aetiology of pneumonia [38].
3 We included fever to help us distinguish pneumonia and asthma syndrome. Fever is more likely to be present in children with pneumonia compared to those with asthma syndrome [28].

Audible wheeze refers to a high-pitched whistling sound heard without any diagnostic aid, when the child breathes out. It can be heard by the caretaker or clinician. An auscultatory wheeze is a high-pitched sound heard with the aid of the stethoscope when the child breathes out [19].

### Diagnosis of asthma syndrome and pneumonia

A panel of three pediatricians with experience in pediatric pulmonology and infectious diseases reviewed the case report form for each participant. They (paediatricians) had no access to the children and hence evaluated the findings post hoc. Each expert reviewed the case report individually and guided by the study definitions, made a diagnosis. During the panel discussions, each expert supported their choice of diagnosis. A diagnosis was considered final if all or two of the three panelists were in agreement. Where there was discordance among the three panelists, a thorough case discussion followed until a final diagnosis was assigned. Each participant was assigned a diagnosis (asthma, bronchiolitis, bacterial pneumonia, viral pneumonia, combination of asthma and bacterial pneumonia, others). A participant had to obtain a minimum score of 75% of the criteria to be assigned the corresponding diagnosis (table 1). 

**Table 1:** Study definitions for asthma, bronchiolitis and pneumonia.

**Diagnosis**	**Criteria**
**Asthma**: Highly probable if 4 of 5 are present	1) Cough, wheeze, difficulty in breathing (**at least one**)
	2) Recurrent cough, wheeze and/or difficulty in breathing, positive history of atopy in child (eczema, rhinitis, food, conjunctivitis), history of asthma in first degree relative (**at least one** )
	3) Fast breathing, chest indrawing, prolonged expiration, rhonchi (**at least 3**)
	4) Good response to bronchodilators
	5) Chest x-ray: normal or hyperinflation
**Bronchiolitis**: Highly probable if 1 and any other criteria are present	1) Age less than 2 years, cough, difficulty in breathing, index episode of wheeze (**all must be present**)
	2) Fast breathing, prolonged expiration, chest indrawing, rhonchi (**at least two**)
	3) Total white cell count ≤15x10^9^cells/l, CRP < 40mg/l, positive RSV (**at least one**)
	4) Chest x-ray: normal or hyperinflation
**Bacterial pneumonia**: Highly probable if 4 of 5 are present	1) Fever, cough, difficulty in breathing (**at least two**)
	2) Axillary temperature ≥38°C, fast breathing, chest indrawing (**at least 2**)
	3) CRP ≥40mg/l, total white cell count ≤ 15 x10^9^ cells/l, Neutrophils ≥65% (**at least one**)
	4) Positive blood culture
	5) Chest x-ray: alveolar infiltrates, consolidation, pleural effusion (**at least one**)
**Viral pneumonia**: Highly probable if 3 of 4 are present	1) Fever, cough, difficulty in breathing (**at least one**)
	2) Axillary temperature ≥38°C, fast breathing, chest indrawing (**at least 2**)
	3) CRP <40mg/l, total white cell count <15 x10^9^ cells/l, lymphocytes ≥45% , positive RSV (**at least one**)
	4) Chest x-ray: normal or diffuse infiltrates

CRP- C-reactive protein, RSV-Respiratory Synctial Virus

### Statistical considerations

To determine the proportion of children with asthma syndrome and pneumonia among children with cough and/or difficult breathing, a minimum sample size of 408 was calculated. We assumed a power of 90% with 95% confidence level, and the proportion of children with asthma syndrome to be 46% based on a study by Sachdev and colleagues [3]. However, this was part of a larger study involving 614 children and all were included in the analysis.

 Data was double-entered in Epidata version 3.0 and exported to Stata version 12.0 (Stata Corp, College station Texas, USA) for analysis. A description of the participants’ characteristics was done using frequency tables, proportions and confidence intervals as appropriate. Logistic regression analysis was used to determine clinical characteristics that were independently associated with asthma syndrome or pneumonia. Cohen’s kappa was used to measure degree of agreement between the primary radiologists. 

## Results

### Description of participants

From August 2011 to June 2012, we recruited 614 children with cough and/or difficulty in breathing. The median age was 10 months (Inter-quartile range 6-18 months) and 80.3% (493 of 614) were aged 24 months and below. There were 347 (56.5%) males. Twenty eight (4.6%) children were born prematurely, 10 (35.7%) of whom had asthma syndrome. Three (30%) of the 10 preterms with asthma syndrome tested positive for RSV. None of the preterms had ever received Palivizumab. Of the 460 (84.7%) children who were reported to have received the complete dose [39] of pentavalent vaccine (DPT – Hepatitis B + *Haemophilus influenzae* type b), 178 (38.7%) had bacterial pneumonia.

### Magnitude of asthma syndrome and pneumonia

**Figure 1:**
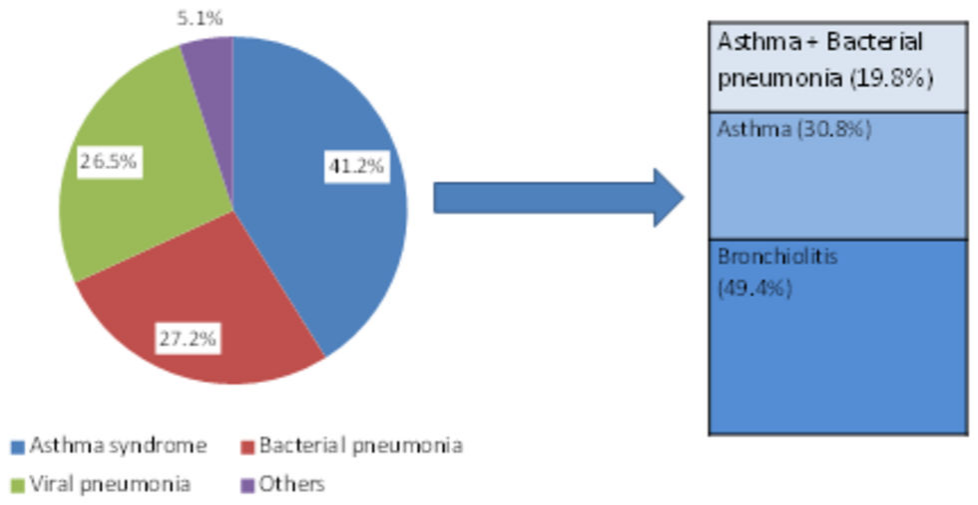
Magnitude of asthma syndrome and pneumonia among ‘under-fives’ in Mulago hospital Uganda.

 Of the 614 children, 41.2% (95% CI: 37.3 - 45.2) had features of asthma syndrome, 27.2% (95% CI: 23.7 - 30.9) had bacterial pneumonia, 26.5% (95% CI: 23.1 - 30.2) had viral pneumonia, while 5.1% (95% CI: 3.5 - 7.1) had other diagnoses including pulmonary tuberculosis and *pneumocystis jirovecii* pneumonia (Figure 1). Of the 253 children with asthma syndrome, 50 (19.8%) had combined asthma and bacterial pneumonia. Only 9.5% of the children with asthma syndrome had been previously diagnosed with asthma. The majority (80.2%) of the children with asthma syndrome were aged 24 months old and below. Of these, 61.6% had bronchiolitis and 38.4% had asthma. 

Overall, 380 of 614 (61.9%) children had pneumonia; 167 (44.0%) had bacterial pneumonia, 163 (43.0) had viral pneumonia and 50 (13%) had combined asthma and bacterial pneumonia. During the panel discussions, discordance among all the three experts occurred in 33 (5.4%) of the 614 cases.

### Clinical and laboratory findings of children with asthma syndrome and pneumonia

Of the 253 children with asthma syndrome, an audible wheeze was appreciated in only 35% (95% CI: 29.7 - 41.8). There was no statistically significant difference in proportion of children with hypoxia between asthma syndrome and bacterial pneumonia (OR 1.1, 95% CI: 0.7 - 1.6, p=0.669) or the children with viral pneumonia (OR 1.2, 95% CI: 1.0 - 1.6, p=0.110). Likewise, there was no statistically significant difference in proportion of children with low peripheral oxygen saturation between asthma syndrome and combined asthma and bacterial pneumonia (OR 1.0, 95% CI: 0.8 - 1.3, p=0.863). 

Even though some laboratory characteristics were part of particular diagnoses, positive results were noted among the different diagnostic categories. For example, 50 (24.6%) of the 203 children with asthma syndrome alone had raised serum C-reactive protein and 18 (8.9%) had raised total white cell counts. The proportion of children with positive RSV test was similar in all diagnostic categories (table 2).

**Table 2:** Summary of clinical and test results of children with asthma syndrome and pneumonia.

**Characteristic/Test**	**Asthma Syndrome**			**Bacterial Pneumonia**			**Asthma +Bacterial Pneumonia**			**Viral Pneumonia**		
	Total	Positive	%	Total	Positive	%	Total	Positive	%	Total	Positive	%
Axillary temperature ≥38°C	203	43	21.2	167	71	42.5	50	18	36.0	163	42	25.8
Audible wheeze	203	72	35.5	167	2	1.2	50	18	36.0	163	5	3.1
Raised *WBC (15X10^9^ cells/l)	199	18	9.1	166	70	42.2	48	17	35.4	160	20	12.5
Raised Neutrophils (≥65%)	199	15	7.5	166	57	34.3	48	31	64.6	160	7	4.4
Raised lymphocytes (≥45%)	199	104	52.3	166	24	14.5	48	3	6.3	160	88	55.0
Raised †CRP (≥40mg/dl)	193	50	25.9	156	116	74.4	48	34	70.8	151	55	36.4
Positive blood culture	203	0	0.0	165	18	10.8	50	6	12.0	163	0	0.0
RSV positive	203	39	19.2	164	20	12.2	50	9	18.0	159	29	18.2
Positive blood smear for malaria parasites	203	52	25.6	167	39	23.4	50	9	18.0	162	51	31.5
≠SaO_2_ (<92%)	203	98	48.3	167	90	53.9	50	30	60.0	163	69	42.3
Abnormal chest x-ray	196	77	39.3	159	127	79.9	48	45	93.8	158	51	32.3

*Whitecellcount,†C-reactiveprotein,≠peripheraloxygensaturation

***Wewereunabletocarryoutsometestsonsomeofthechildren,hence,thevariationinthetotalnumberofchildrenforeachofthetests***.

 Overall, 26.4% (95% CI: 22.9 - 30.1) of the children had a positive blood smear for malaria parasites, most of whom (31.5%) had viral pneumonia. HIV testing was performed on 589 (95.9%) of the children and of these, 41(7%) were found to be HIV positive (95% CI: 5.0 - 9.3). The primary radiologists agreed on the chest x-ray findings in 79.4% of the cases (Cohen’s kappa = 0.72, SD = 0.03, p = 0.000). 

### Medication for children with asthma syndrome and pneumonia

A total of 599 (97.6%) of the study participants had prescriptions for antibiotics. Of the 253 children with asthma syndrome, 95.3% (95% CI: 91.9 - 97.5) had a prescription for antibiotics, 87.7% (95% CI: 83.1 - 91.5) for short-acting β_2_ agonists (SABA) and 43.1% (95% CI: 36.9 - 49.4) for systemic steroids. However, of these, 50 (19.8%) had bacterial pneumonia and their antibiotic prescriptions were justified. 

## Discussion

In this paper, we highlight the findings of magnitude of asthma syndrome and pneumonia among children aged 2 to 59 months attending Mulago National referral and teaching hospital. Among young children, it is difficult to distinguish asthma from bronchiolitis because of the similarities in aetiological and risk factors as well as clinical presentation [12,17,29,40]. Several terminologies such as asthma, bronchiolitis, bronchitis- asthma, hyper-reactive airways disease and wheezing disorder are used to describe children who present with wheezing [12]. In a study on the same sample of children, we noted similarities in factors associated with asthma and bronchiolitis, suggesting common aetiological and risk factors [29]. Other studies have also alluded to the challenges of distinguishing asthma from bronchiolitis among young children [41]. In this study, we combined children with asthma and bronchiolitis and referred to them as “asthma syndrome” to include all children with cough and wheezing because these symptoms are treatable. Furthermore, using the term asthma indicates the potential for follow up and identification of children who have asthma for appropriate management. 

### Magnitude of asthma syndrome

According to the study criteria, 41.2% of the children had features of asthma syndrome. This indicates that asthma syndrome is a significant cause of cough and/or difficulty in breathing in children less than five years of age. These findings are similar to those by Sachdev and colleagues who studied 200 children aged 6-59 months with WHO-defined severe pneumonia and wheezing, and noted that 46% of them had asthma [3]. Using a similar criterion, Hazir and colleagues found a high prevalence (49%) of asthma among 1622 children aged 1-59 months in Pakistan [42]. Other studies defined asthma based on previous history of cough and difficulty in breathing, and current/previous history of audible wheeze, an approach that excluded children in whom audible wheeze was not recognized [3,43]. Having used the approach of thorough clinical assessment, laboratory and radiological test results as well as response to medication, to diagnose asthma syndrome, we were able to identify children who would otherwise have been missed if the definition of asthma syndrome was based on presence of only audible wheeze. Therefore, the estimates from this study are fairly accurate.

### Magnitude of pneumonia

Overall, 61.9% of the participants had pneumonia, of whom, more than half had bacterial pneumonia. These findings are comparable to earlier studies in low-income settings [23,25,44]. Estimates by WHO show that more than 90% of acute lower respiratory tract infections in low-income countries are classified as pneumonia, and the majority are presumed to be bacterial [1,45]. The differences between the findings in our study and WHO estimates may be attributed to differences in case definitions for pneumonia. According to WHO, pneumonia is defined as presence of cough and/or difficulty in breathing in association with fast breathing, with/without lower chest in-drawing whereas in this study, other clinical findings and test results were also considered (table 1). The systematic approach of history, clinical signs and tests may have increased specificity. These findings reiterate current arguments that, using WHO guidelines, bacterial pneumonia is over-diagnosed.

 Viral pneumonia was present in 26.5% of the participants. Even though severe pneumonia has been mainly associated with bacterial etiology [46], evidence from studies in low-income countries shows that viral infections are responsible for a significant proportion of severe pneumonia among young children[25,34,47]. In low-income countries like Uganda, many children present with co-morbidities such as malaria and malnutrition and this may exacerbate the severity of viral pneumonia. However, in this study, there was no significant association between viral pneumonia and a positive malaria test. Similarly, only 6.8% of the children with viral pneumonia had severe acute malnutrition. Hence, we were unable to explain the reasons for the high proportion of children with viral pneumonia in this study. Further studies on viral pneumonia are recommended to contribute towards deeper understanding on the magnitude, spectrum and significance of viruses in causation of pneumonia.

### Clinical and laboratory characteristics of the children with asthma syndrome and pneumonia

There were many similarities in test results between asthma syndrome and viral pneumonia. This may suggest a common primary cause which is a viral illness. The literature shows that asthma exacerbations and bronchiolitis are commonly triggered by respiratory viral infections [12]. Likewise, viral pneumonia may start as an upper respiratory viral infection which spreads to the lung parenchyma causing pneumonia [23]. In some cases, children with viral pneumonia may have signs and symptoms that are similar to those of asthma syndrome such as wheezing, preceded by an upper respiratory tract infection [48]. The similarities indicate the possibility that children who were categorized as viral pneumonia could have had asthma syndrome, a hypothesis which needs to be explored in future research.

Viral respiratory infections have been associated with asthma exacerbations in up to 80% of cases [49]. Similarly, viruses are the major cause of bronchiolitis in children. In this study, the proportion of children with positive RSV test was less than 20% and its frequency was similar in all diagnostic categories. RSV infection has been considered a major trigger of asthma exacerbations and, an important cause of bronchiolitis and viral pneumonia among young children [23,50], but other viruses also play a role. We did not test for other respiratory viruses. Therefore, we are unable to document the association between respiratory viruses and exacerbations/causation of asthma syndrome. Further research on viruses and asthma syndrome is recommended, especially in view of the current findings of high magnitude of children with asthma syndrome.

Overall, a quarter of the study participants had malaria. Of these, 72% had pneumonia as well. Research has consistently documented the overlap in clinical presentation of malaria and pneumonia among children [51,52]. In addition, the IMCI case definitions for malaria and pneumonia are not very specific [53]. Our findings add to the current evidence of overlap in clinical presentation of malaria and pneumonia. There is need to refine the IMCI case definitions for malaria and pneumonia to improve on management of both conditions, reduce irrational antibiotic use and contribute towards reduction of mortality among children less than five years of age.

Blood cultures for identification of fastidious bacteria were done in 612 children, and a pathogen was identified in only 3.9% of them. These findings show a much lower proportion of bacteraemia compared to earlier studies which were done in the same setting [6,7]. This may be attributed to differences in study population. These studies focused on children with WHO severe pneumonia and excluded those with wheezing [6,7]. In addition, 97% of the participants in the current study had a history of having been given antibiotics before presenting to the hospital. This is in contrast to the findings in the previous study [6] where half of the children had history of having received antibiotics prior to getting to the hospital. The overwhelming use of antibiotics in the current study population may have reduced the probability of identifying bacteria in blood.

### Medication for children with asthma syndrome and pneumonia

The majority (95.3%) of the children with asthma syndrome had a prescription for antibiotics. However, only a small proportion (19.8%) of them had bacterial pneumonia and the prescription of antibiotics was justified. About 12% of the children with asthma syndrome had no prescription for short-acting β_2_ agonists (SABA). In more than half of the children with asthma syndrome, systemic steroids were not prescribed. These findings confirm the earlier reports which indicated that, among children less than five years presenting with acute respiratory symptoms, bacterial pneumonia is over-diagnosed, contributing to irrational use of antibiotics and asthma under-diagnosed, and therefore asthma medicines under-utilized [3,54]. In addition, these findings point to the possibility that even in tertiary care settings where more skilled healthcare providers exist, clinicians tend to use primary care guidelines for diagnosis of bacterial pneumonia. Further research into the clinical practice regarding acute respiratory illnesses in tertiary care settings is recommended.

## Methodological Considerations and Study Limitations

### Study definition for asthma and pneumonia

 Previous researchers and clinical guidelines have used audible wheeze to diagnose asthma [13,43]. However, audible wheeze has low sensitivity [3,14,42]. Similarly, many earlier studies used WHO case definition of pneumonia, an approach that leads to over-diagnosis of pneumonia [3,4]. Chest x-ray findings have also been used to define pneumonia. However, chest x-rays have very low sensitivity for diagnosis of pneumonia [55]. We used a strict criteria based on history, clinical examination and test findings as well as response to treatment, to define asthma and pneumonia in this study population. Hence, this approach provided fairly accurate estimates of the magnitude of asthma and pneumonia among children less than five years of age in Mulago hospital Uganda.

### Diagnostic approach

We used strict study definitions that were based on history, clinical examination, laboratory tests, radiological findings and response to treatment, to aid diagnosis. The chest radiographs were interpreted by two independents radiologists. In addition, we subjected the findings for each of the study participants to a review by a panel of three experts. Therefore, we believe that this process increased the accuracy of the diagnoses hence providing more accurate estimates. However, the experts had no access to the study participants and reviewed the case report forms post hoc. Therefore, their discussions on the clinical findings depended entirely on the record by the study doctor. Any errors that were made when examining the patients could not be corrected and may have resulted into misdiagnosis of some children. However, such errors are presumed to be very minimal because the study doctors were trained and closely supervised by the principal investigator. 

Even though we used systematic and stringent diagnostic criteria, there may be some children who were misclassified as pneumonia yet had asthma syndrome and vice versa. Similarly, there may be children who had bacterial pneumonia but were classified as viral pneumonia and vice versa. We were unable to estimate the degree of error because of lack of diagnostic gold standard for asthma syndrome and pneumonia among children less than five years of age. However, given the extensive classification methods that we used, the errors are presumed to be minimal and hence the resulting estimates fairly accurate. 

 Finally, this study was done in a tertiary hospital. The findings may only be applicable to hospitals with similar settings and not to lower level units or the general population.

## Conclusions, Recommendations and Perspectives

Asthma syndrome causes significant acute respiratory morbidity among children less than five years of age, contrary to previous reports from Uganda that pneumonia is almost the sole cause of acute respiratory illnesses in this age group. This implies that children with asthma syndrome have been inappropriately managed as pneumonia. Antibiotics are used irrationally due to mis-diagnosis of asthma syndrome and viral pneumonia as bacterial pneumonia. Children with asthma syndrome who are misdiagnosed miss out on short and long term care which may impact on their growth and development and, overall quality of life. This has implications on healthcare costs to the families and healthcare systems and, childhood morbidity and mortality. Research on management of children with asthma syndrome is needed. There is need for diagnostic tools that can be used to differentiate asthma syndrome and pneumonia at all levels of healthcare. The findings also point to the need for review of the current WHO case definitions for asthma and pneumonia. Finally, a study to understand reasons for under-diagnosis of asthma syndrome among children less than five years in Mulago hospital is recommended. 

Asthma symptoms may be chronic or recurrent and usually start in infancy. The symptoms persist in about 30-50% of the affected children [33]. Over time, many terminologies have been used to describe asthma symptoms such as wheezing disorder, bronchitis and bronchitis asthma [12]. However, the role of symptom control among these children cannot be under-estimated. It is important to think about using pragmatic terms such as asthma or asthma syndrome among children with asthma symptoms for purposes of treatment. Studies and consensus on definitions, diagnostic terms and management of asthma symptoms among children less than five years of age are recommended.
